# The benefits of providing absolute rather than relative risks: evidence from a naturalistic study with the BBC

**DOI:** 10.1098/rsos.261216

**Published:** 2026-11-11

**Authors:** George Farmer, Alexandra Freeman, David Spiegelhalter, William Skylark

**Affiliations:** ^1^ Andrew Mayes Centre for Cognitive Neuroscience, The University of Manchester, Manchester, UK; ^2^ Winton Centre for Risk and Evidence Communication, Department of Pure Mathematics and Mathematical Statistics, University of Cambridge, Cambridge, UK; ^3^ Hertford College, University of Oxford, Oxford, UK; ^4^ Department of Psychology, University of Bath, Bath, UK

**Keywords:** relative risks, absolute risks, science communication, judgement, decision-making, health, risk communication

## Abstract

Health stories in the media often contain relative risks, for example, ‘Eating bacon increases your risk of colorectal cancer by 20%’. While the reported changes in risk may be accurate, they do not allow the reader to know the relevant absolute risks (i.e. their chances of developing cancer if they do, or do not, eat bacon). We investigated what people infer the absolute risks to be when they are only given relative risks. Visitors to the British Broadcasting Corporation (BBC) news website read a story about the protective effects of mushroom consumption against mild cognitive impairment. Participants either saw the change in risk presented in relative format (50% decrease) or absolute format (from 19 per 100 to 9 per 100). Over 1000 people took part in the study and their data were analysed according to a pre-registered plan. We found that the median estimates of the absolute risks were correct even when participants only saw relative information, indicative of a ‘wisdom of the crowd’ effect. However, the relative format led people to have much larger errors in their estimates of the true risks when compared to the absolute format. Furthermore, the absolute format did not reduce participants' self-reported comprehension or interest in the story.

## 1. Introduction

When a member of the public reads in the press that taking ibuprofen results in a 100% increased risk of a heart attack [1], what impression are they left with about how risky ibuprofen is? What do they think their heart-attack risk is if they do not take ibuprofen? And what do they think the risk is if they do take it?

Press release and headline writers frequently opt for relative risks when promoting stories on scientific research, for example: ‘Revealed: Footballers five times more likely to suffer from Alzheimer's disease, landmark study finds’ [2] or ‘Patients with Covid and Flu double the risk of dying, say scientists’ [3]. However, to properly understand the risks involved one needs to know the absolute values. Is the base rate 1 in a 100 or 1 in 10? Knowing this is critical for a person to understand whether they should take action to reduce the risk. If the base rate is very low, an increase might be negligible, if the base rate is high, an increase could be alarming.

There are probably several factors contributing to the prevalence of relative risks in news stories. One being the need to grab a reader's attention. Relative risks have an advantage in this respect because they often result in a more dramatic sounding change than absolute risks. For example, ‘drug cuts risk by half’ is more dramatic (and probably more memorable) than ‘drug cuts risk by one percentage point’, though both versions could be accurate accounts of the same scientific finding. Another plausible factor is the perception that absolute risks lack concision or clarity. Writing in a story that a drug reduced people's risk of disease from 5 in 100 to 3 in 100 is certainly less concise than the equivalent relative risk, but it is far from certain that the absolute format would reduce comprehension. In fact, previous research has shown that the natural frequency format (e.g. 10 in 100) is generally better understood than the more abstract normalized percentage format of 10% [4].

There is reason to believe that media representations of health risks do influence the public's perception. Lichtenstein *et al.* found that dramatic causes of death were over-represented in the media, whereas less dramatic causes were under-represented [5]. The knock-on effect was that the public perceived dramatic causes of death to be more frequent than they actually were, although an alternative explanation for this finding is simply regression to the mean [6]. By 1997, not much had changed with an analysis of print media showing that common causes of mortality took up much less space than more dramatic causes. For example, tobacco took up just 23% of the space one would expect it to based on its actual contribution to mortality, while illicit use of drugs took up 1740% [7]. The use of relative risks has been highlighted as a common choice in the advertising of drugs to consumers, in their review of medicine marketing Schwartz & Woloshin [8] provide the example of a heart disease drug advertised as reducing deaths from heart attacks by 42%, which the small print revealed to be a decrease in absolute risk from 8.5% to 5%.

In medical settings we know that relative risks can influence behaviour. One study of patients, using hypothetical scenarios, found that 57% of respondents would choose a particular medicine when its benefits were described in relative terms, but only 15% would choose it when the benefits were expressed in absolute terms [9]. The same study also found that when presented with the absolute base rate and the relative change, only 28% of respondents correctly identified the absolute benefit provided by the medicine. In a study with both doctors and patients, participants were asked whether a new drug that reduced mortality, but with more side effects than existing drugs, was effective [10]. Of doctors who were provided with an absolute reduced mortality of 2 percentage points, 68% agreed the drug was effective. When the same change in mortality was expressed as a relative reduction, 94% of doctors agreed the drug was effective. The proportions were similar for patients with 67% agreeing under absolute presentation and 90% agreeing under relative presentation.

Although a relative risk does not allow someone to infer the absolute risk, the reader of a relative risk is not totally without information. They are likely to assume a base rate given their experience of the topic in question. This will of course mean that people's perception of the true risks will vary widely with differing levels of expertise. Consistent with this, research on decision-making shows that people often infer plausible values when relevant information is omitted from a problem description [11,12]. Even if a person does accurately estimate the relevant base rate, they still need sufficient numeracy to understand what a 50% increased risk means. This could be correctly interpreted as an increase from 10% to 15%, or it could be misinterpreted as an increase from 10% to 60%, that is, an increase of 50 percentage points. As noted above, research suggests that even when provided with an absolute base rate and a relative change many people do not correctly infer the resulting new absolute risk [9].

For understandable reasons, much of the existing work on relative risks in medical settings concerns hypothetical situations, whereas work on the media's impact on risk perception tends to be observational rather than experimental. By contrast, in the experiment outlined below, we manipulated the risk format used in a genuine live British Broadcasting Corporation (BBC) news story about a health risk [13]. Readers were randomized to see either a relative change format or an absolute change format. The experiment allowed us to test the effect of the absolute versus relative format on several metrics including recall, inferred absolute risks, comprehension and interest.

## 2. Material and methods

We took a real press release and worked with the BBC to produce two stories that were identical except for the risk format, which was either absolute or relative. Visitors to the BBC News website between 14.30 GMT on 14 March 2019 and 17.00 GMT on 15 March 2019 were randomized to see either the relative or absolute story. At the end of the story, there was a link inviting readers to complete our survey.

The study received ethical approval from the University of Cambridge Psychology Research Ethics Committee (PRE.2018.005). Before inspection of the data, we pre-registered our analysis plan including participant exclusion criteria: https://osf.io/mg42z/.

### 2.1. Design and procedure

Participants were directed to a survey with questions about the story they had just read. Our first independent variable was story type, which varied the risk format (absolute or relative) used in the story on the BBC news site. In the absolute risk format, participants read that ‘eating mushrooms lowered the chances of mild cognitive impairment, so that roughly nine out of 100 people who ate more than two portions a week were diagnosed, compared with 19 out of 100 among those who ate fewer than one portion’. In the relative risk format participants read that ‘that eating more than two portions of mushrooms a week lowered the chances of mild cognitive impairment (MCI) by 50%, compared with those who ate fewer than one portion’.

Our second independent variable was task (recall or estimation). Participants in the recall group were simply asked to restate the risk in the same format that they had read it. Participants in the estimation group were asked to state the risk in absolute format, if they had read the story in the relative format. They were asked to state the risk in a relative format, if they had read the story in the absolute format. Figure 1 outlines the experiment design and randomization procedures.

**Figure 1.**
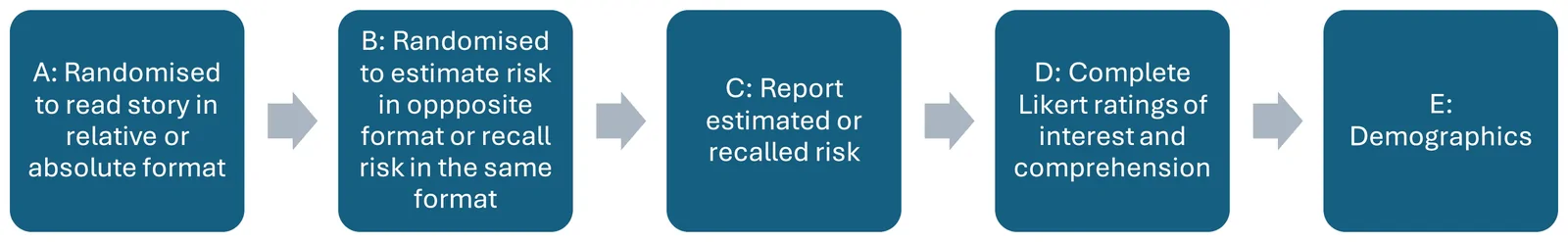
Study flowchart illustrating the order of tasks and randomization steps. The order of steps C and D were counterbalanced. Within step D the order of interest and comprehension ratings was also counterbalanced. Demographic questions were always last.

Participants were also asked to rate the story in terms of interest and ease of understanding on a seven-point Likert scale ranging from ‘completely disagree’ to ‘completely agree’. We randomized whether participants completed the rating task first or the risk reporting question first. Our dependent variables were the values that participants reported in response to the risk questions, and their interest and comprehension ratings.

### 2.2. Participants

Participants consisted of visitors to the BBC news website who read the story ‘Mushrooms may “reduce the risk of mild brain decline”’ [13,14] and then clicked on a link at the end of the story inviting them to take part in a study.

There were 1691 participants who started the study. After exclusions for incomplete responses (539), being under 18 years old (one) and having a duplicate IP address (103), a total of 598 exclusions were made (some participants fell under multiple exclusion categories; details of the exclusion criteria are in the pre-registration document). A total of 1093 participants were included in the analysis; 58.01% of participants reported being female, 41.72% male and 0.27% reported ‘other’. The mean age was 51.82 (s.d. = 15.08) with a range of 18–91. See table 1 for education and English language proficiency.

**Table 1.** Participant demographics. (Breakdown of participants self-reported education and English language proficiency.)

	*n*	%
education		
higher degree	445	40.71
bachelor's degree	435	39.80
secondary school	198	18.12
none	15	1.37
language proficiency		
mother tongue	974	89.11
fluent	104	9.52
intermediate	13	1.19
beginner	2	0.18

As shown in table 2, there were more participants who had read the absolute story than the relative story. Because the absolute story was the default story on the BBC news website, only readers whose browser settings allowed for it could be randomized to the relative story. Therefore, more readers were served the absolute story than the relative story.

**Table 2.** Participants per condition. (Number of participants assigned to each of the four conditions. Additionally, broken down by whether participants completed the interest and comprehension ratings first or the task first.)

	risk estimation task		risk recall task	
story	rating first	rating second	rating first	rating second
absolute	162	172	158	155
relative	101	88	115	142

## 3. Results

Participants took a median time of 96 s to complete the survey and a Wilcoxon rank-sum test found there was no significant difference between the absolute (Mdn = 95 s) and relative (Mdn = 99 s) stories on this measure, W=137,933, *p* = 0.216.

### 3.1. Accuracy of recall

To determine whether people could remember the numbers they read about we analysed the accuracy of their responses. The distributions of recall responses for each condition are plotted in figure 2, with the corresponding descriptive statistics reported in table 3. In all cases, the median and mode of the response distribution both coincide with the correct response. As is clear from figure 2, the responses were markedly non-normal. Our pre-registration stated: ‘We will also use non-parametric Wilcoxon tests to check that findings are robust to any violations of the *t*-test normality assumption’. Correspondingly, here and in the sections that follow we report both one-sample *t*-tests and Wilcoxon tests for each estimate; the results are reported in table 3.^1^

**Figure 2.**
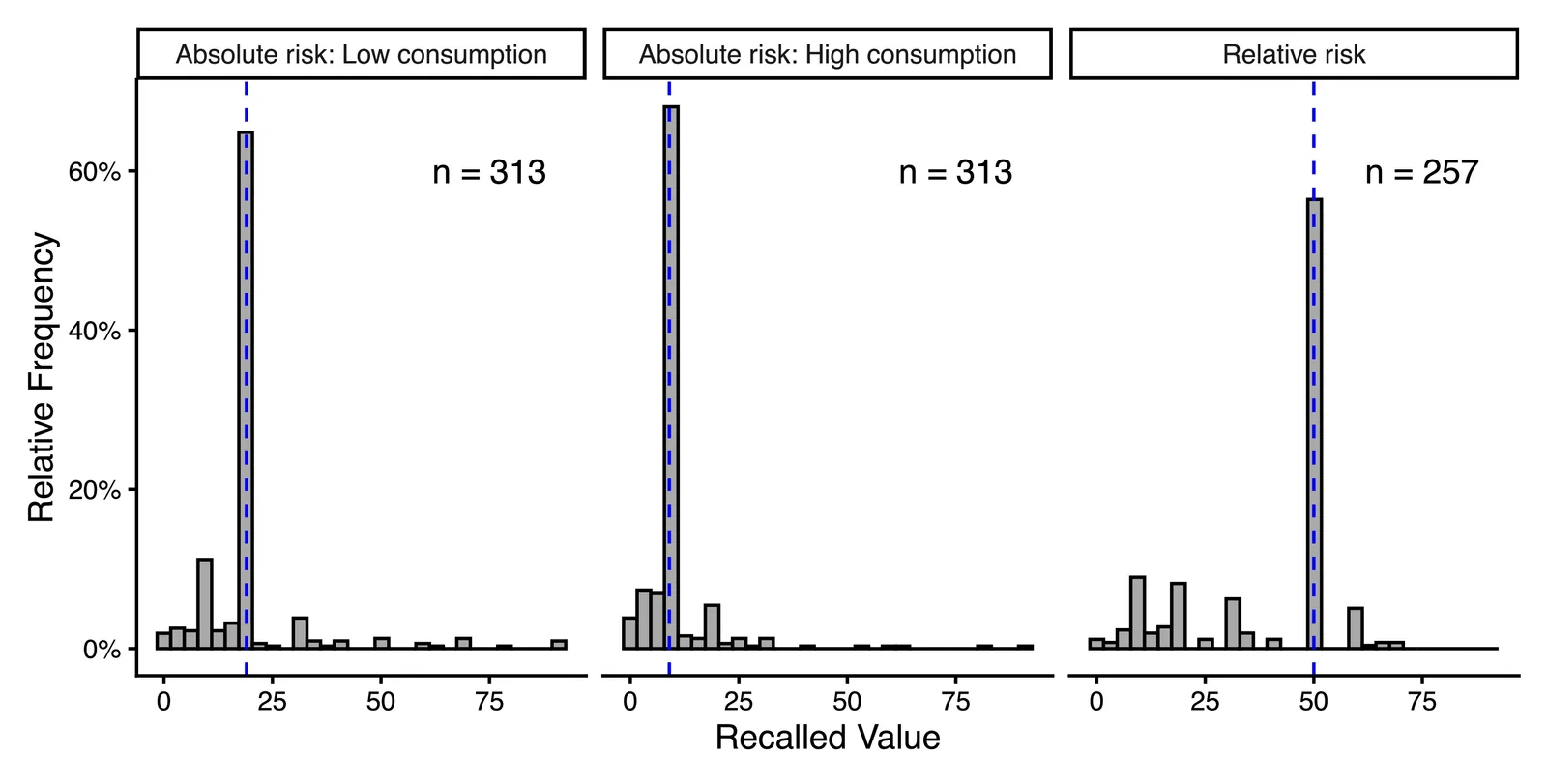
Distributions of participants’ recalled values. In each case, the mode and median are the same as the correct value (show by the dashed vertical line).

**Table 3.** Accuracy of participants’ recalled values. (Table shows the median and mean of the values that participants recalled having read. Wilcoxon and *t*-tests compare recollected values with true values in the story. Pre-registered Bonferroni-corrected significance level = 0.017.)

					Wilcoxon		*t*-test	
story	value in story	*n*	median response	mean response (s.d.)	*V*	*p*	*t* (d.f.)	*p*
absolute	19 in 100	313	19	19.87 (12.86)	3703	0.312	1.20 (312)	0.233
	9 in 100	313	9	10.41 (9.36)	4924.5	0.371	2.67 (312)	0.008
relative	50%	257	50	39.15 (17.45)	238	<0.001	−9.97 (256)	<0.001

In the absolute risk condition, when participants estimated the low mushroom consumption risk, the *t*-test suggests no reason to reject the hypothesis that the mean response is correct, and the Wilcoxon test likewise gives no reason to reject the hypothesis that the data are symmetrically distributed about the correct response value. When participants estimated the low risk, the Wilcoxon test again indicates no reason to reject the hypothesis that the data are symmetrically distributed about the correct value, but the *t*-test indicates a tendency to slightly over-state the risk.

For participants in the relative risk condition, although both the median and mode of the response distributions were again correct, the *t*-test indicates that the mean response is below the true value and the Wilcoxon test likewise indicates rejection of the hypothesis that the responses are symmetrically distributed about the correct response; inspection of figure 2 indicates that there were substantially more responses below the correct value (*n* = 94) than above it (*n* = 18).

Taken together, these results suggest that our participants were generally quite accurate in recalling the numeric values with which they were presented, but with some tendency overstate the smallest value and to understate the largest one—a pattern of results consistent with the general central tendency of judgements under uncertainty, especially when (as here) the response scale is bounded [15,16].

### 3.2. Accuracy of estimation

We sought to determine whether people could accurately estimate absolute risks if they had only read a relative risk. To do this, we compared the estimates from participants who read the relative story with the true values. Figure 3 shows the distribution of estimates. The corresponding descriptive and inferential statistics are shown in table 4.

**Figure 3.**
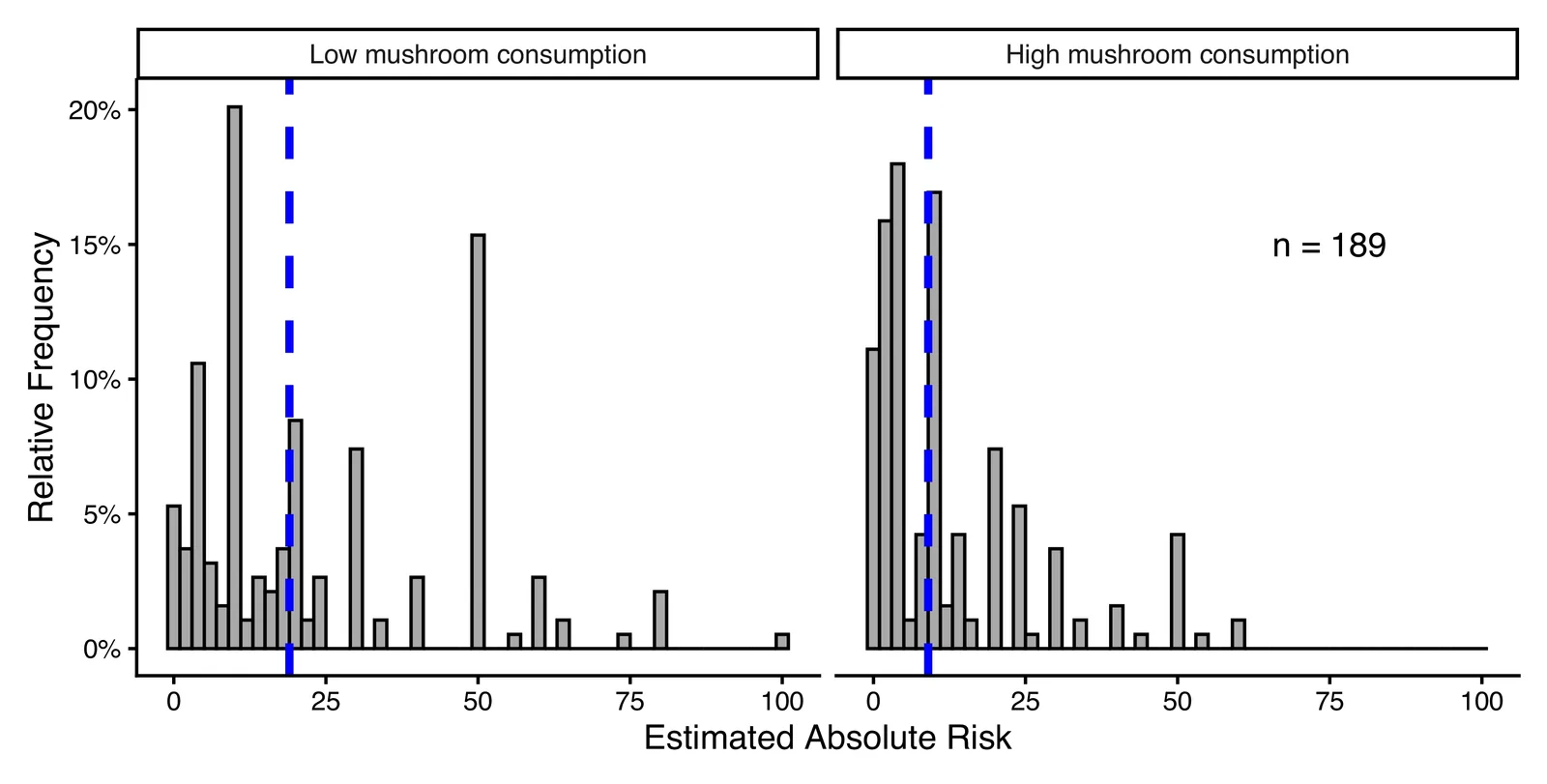
Distributions of participants’ estimates of the absolute risks. Made by participants who had read the relative risk story. The vertical dashed lines indicate the true absolute risks of 19 and 9 per 100. For the high absolute risk (low mushroom consumption) the median estimate was 17, and for the low absolute risk (high mushroom consumption) the median estimate was 9.

**Table 4.** Accuracy of participants’ estimates. (Table shows the median and mean of participants’ estimates of the absolute risks when the story only provided relative risks. Wilcoxon and *t*-tests compare estimated risks with true absolute risks. *n* = 189, pre-registered Bonferroni-corrected significance level = 0.025.)

			Wilcoxon		*t*-test	
true value	median estimate	mean estimate (s.d.)	*V*	*p*	*t* (d.f.)	*p*
19	17	23.62 (20.78)	9385.5	0.177	3.06 (188)	0.003
9	9	12.74 (13.68)	9342	0.197	3.75 (188)	<0.001

The Wilcoxon tests suggest no reason to reject the hypothesis that the estimates are symmetrically distributed about the true values, and the median estimates (9 and 17) are close to the true values (9 and 19). By contrast, the *t*-tests indicate that the mean responses are higher than the true values for both the low risk and the high risk. Like for the recall responses, this probably partly reflects a boundary effect (there is more scope for overestimation than underestimation when the true value is small and the scale is bounded at zero, so even if the response distribution is centred on the true value, the mean will be too large).

### 3.3. Are recollection and estimation different?

We assessed how remembered absolute risks differ from estimated absolute risks. We compared the recollected absolute risks from participants who read the absolute story (see table 3) with the estimated absolute risks from participants who read the relative story (see table 4), with a pre-registered alpha level of 0.025. For both the high and low absolute risks, we found no significant differences between the ‘estimate’ and ‘recall’ conditions (for the low risk, Wilcoxon test *W* = 29 342, *p* = 0.878, independent samples *t*(295.12) = −2.06, *p* = 0.040; for the high risk, *W* = 29 770, *p* = 0.900, independent samples *t*-test *t*(276.02) = −2.24, *p* = 0.026).

### 3.4. Are the size of estimation and recall errors different?

In order to estimate the effect that story format had on the precision of people's estimates, we compared the errors that people made when recalling the absolute risks with the errors made when estimating the absolute risks. To calculate the error, we took the absolute deviation of participants’ responses from the true value.

Table 5 shows the median and mean absolute errors made by participants broken down by the risk format of the story they read, along with the results of Wilcoxon signed rank tests and independent samples *t*-tests comparing the absolute and relative conditions. Both the Wilcoxon and *t*-tests indicate that the absolute errors were smaller for the participants who were recalling the risks than for those who were inferring them from the relative risk information.

**Table 5.** Size of estimation and recall errors. (Table shows the size of the error when participants who read the relative story estimated the absolute risk, and when participants who read the absolute story recalled the absolute risk. Wilcoxon and *t*-tests compare estimation errors and recall errors. Pre-registered corrected significance level = 0.025.)

	relative story (estimation) error		absolute story (recall) error		Wilcoxon		*t*-test	
risk	Mdn	*M* (s.d.)	Mdn	*M* (s.d.)	*V*	*p*	*t* (d.f.)	*p*
high	11	16.10 (13.89)	0	5.64 (11.58)	10 999	<0.001	−8.69 (347.72)	<0.001
low	6	9.35 (10.64)	0	3.49 (8.80)	12 059	<0.001	−6.38 (340.42)	<0.001

### 3.5. Estimated relative risk

We also asked participants who read the absolute risk story to estimate what the relative risk reduction was. The correct answer to this question was 53%. However, the question was sufficiently ambiguously posed that participants could have interpreted it as asking about the reduction in percentage points from high to low risk. Under that interpretation, the correct answer would be 10% (from 19 per 100 to 9 per 100). Given this ambiguity we did not pre-register our analysis of these data, but figure 4 shows the distribution of responses.

**Figure 4.**
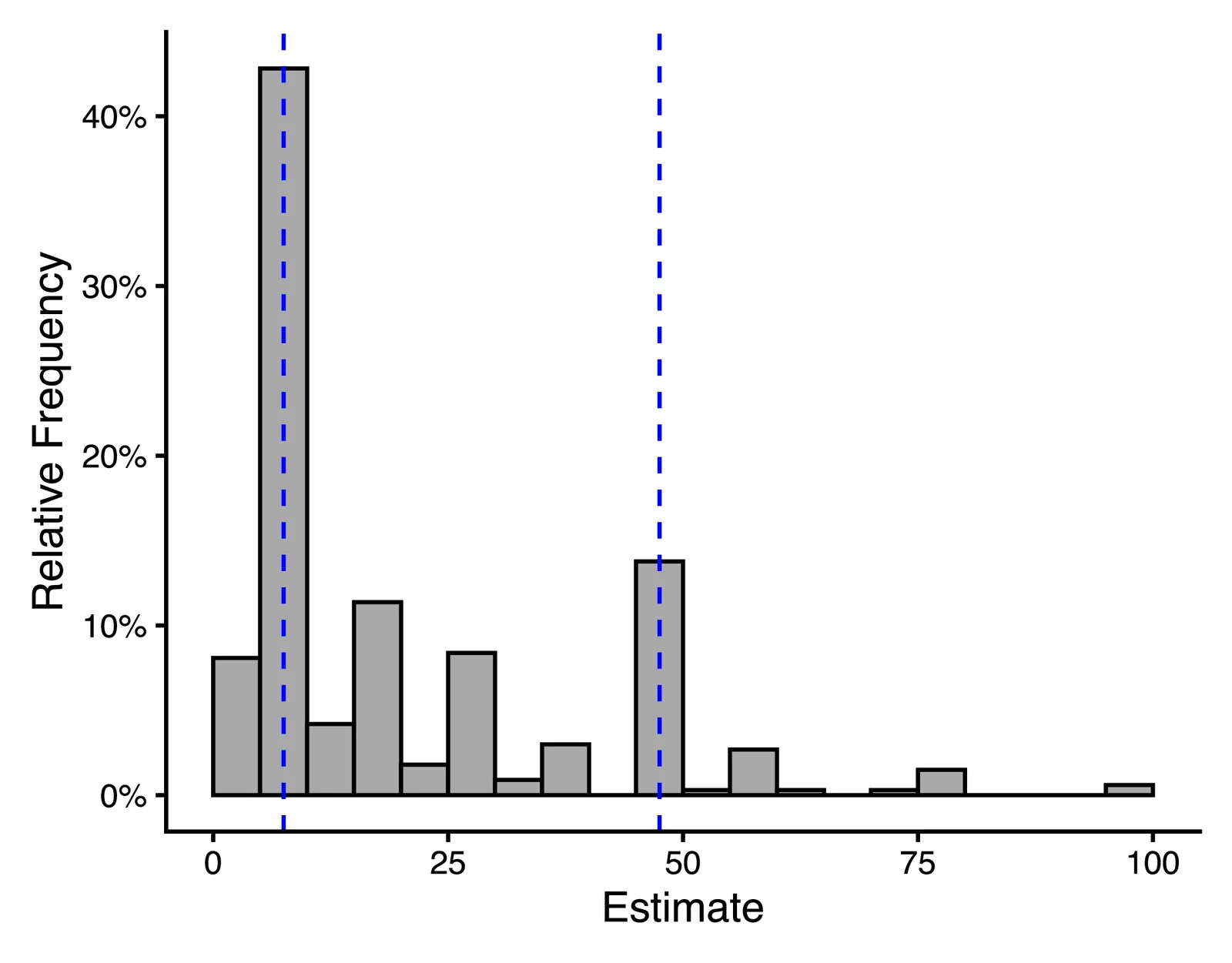
Estimates of the relative risk reduction by readers of the absolute format story. The dashed vertical lines highlight the bins containing the plausible correct responses (10 and approx. 50).

### 3.6. Interest and comprehension

Interest and comprehension were significantly correlated (ρ(N=1093) = 0.50, *p* < 0.001). As per our pre-registration, we analysed these as separate dependent variables because the correlation was below 0.8 (figure 5).

**Figure 5.**
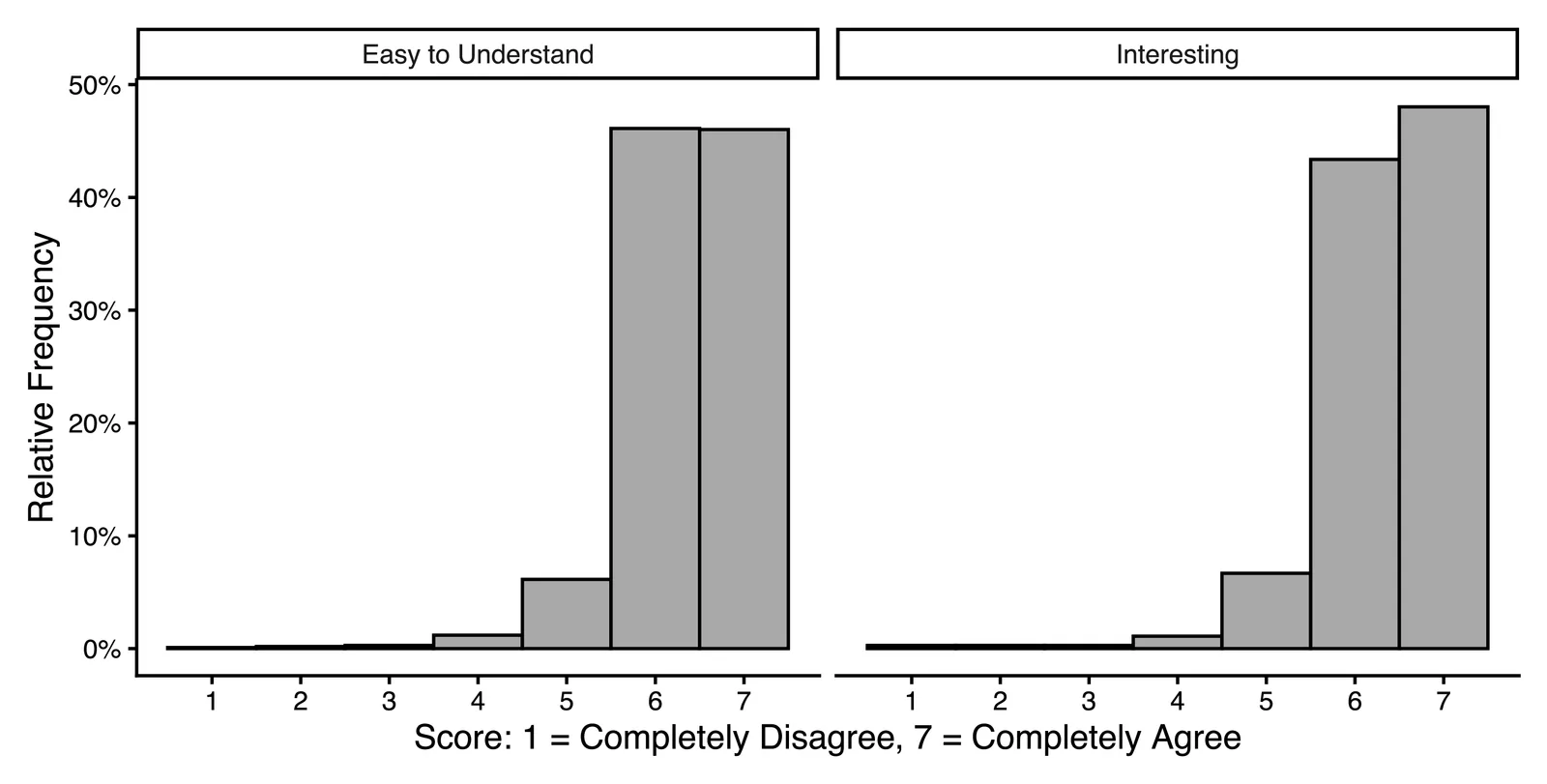
The distribution of participants’ ratings of the stories’ ease of comprehension and how interesting they found them. 1 = completely disagree, 4 = neutral, and 7 = completely agree.

We submitted the interest score to a 2 story type (absolute, relative) by 2 task (recall, estimation) by 2 rating order (first, second) between-subjects ANOVA. The rating order independent variable coded whether participants first rated their interest in the story or first answered the risk questions. Our pre-registered alpha was 0.025 and none of the main effects or interactions reached this level. The ANOVA results are displayed in table 6.

**Table 6.** Interest ratings ANOVA. (Pre-registered, Bonferroni-corrected significance level of 0.025.)

	story	task	rating order	story * task	story * rating order	task * rating order	story * task * rating order
*F*	0.00	1.09	1.72	0.00	2.09	4.06	1.87
d.f.	1,1085	1,1085	1,1085	1,1085	1,1085	1,1085	1,1085
*p*	0.973	0.297	0.190	0.964	0.148	0.044	0.172
$\eta_{p}^{2}$	<0.001	0.001	0.002	<0.001	0.002	0.004	0.002

We repeated the ANOVA with the comprehension rating as our dependent variable, as shown in table 7. There was a main effect of rating order such that comprehension ratings were higher when people completed the rating task first, which was qualified by a two-way interaction with task type, and by a three-way interaction between story type, task type and rating order.

**Table 7.** Comprehension ratings ANOVA. (Pre-registered, Bonferroni-corrected significance level of 0.025.)

	story	task	rating order	story * task	story * rating order	task * rating order	story * task * rating order
*F*	0.03	4.85	19.82	2.49	0.16	14.13	11.51
d.f.	1,1085	1,1085	1,1085	1,1085	1,1085	1,1085	1,1085
*p*	0.855	0.028	<0.001	0.115	0.691	<0.001	<0.001
$\eta_{p}^{2}$	<0.001	0.004	0.018	0.002	<0.001	0.013	0.011

Decomposing the three-way interaction revealed that the effect of task type depended on rating order for the relative story type but not for the absolute story type, *t*(1085) = 4.62, *p* < 0.001 and *t*(1085) = 0.29, *p* = 0.773, respectively. This pattern is clear in figure 6 (top row).

**Figure 6.**
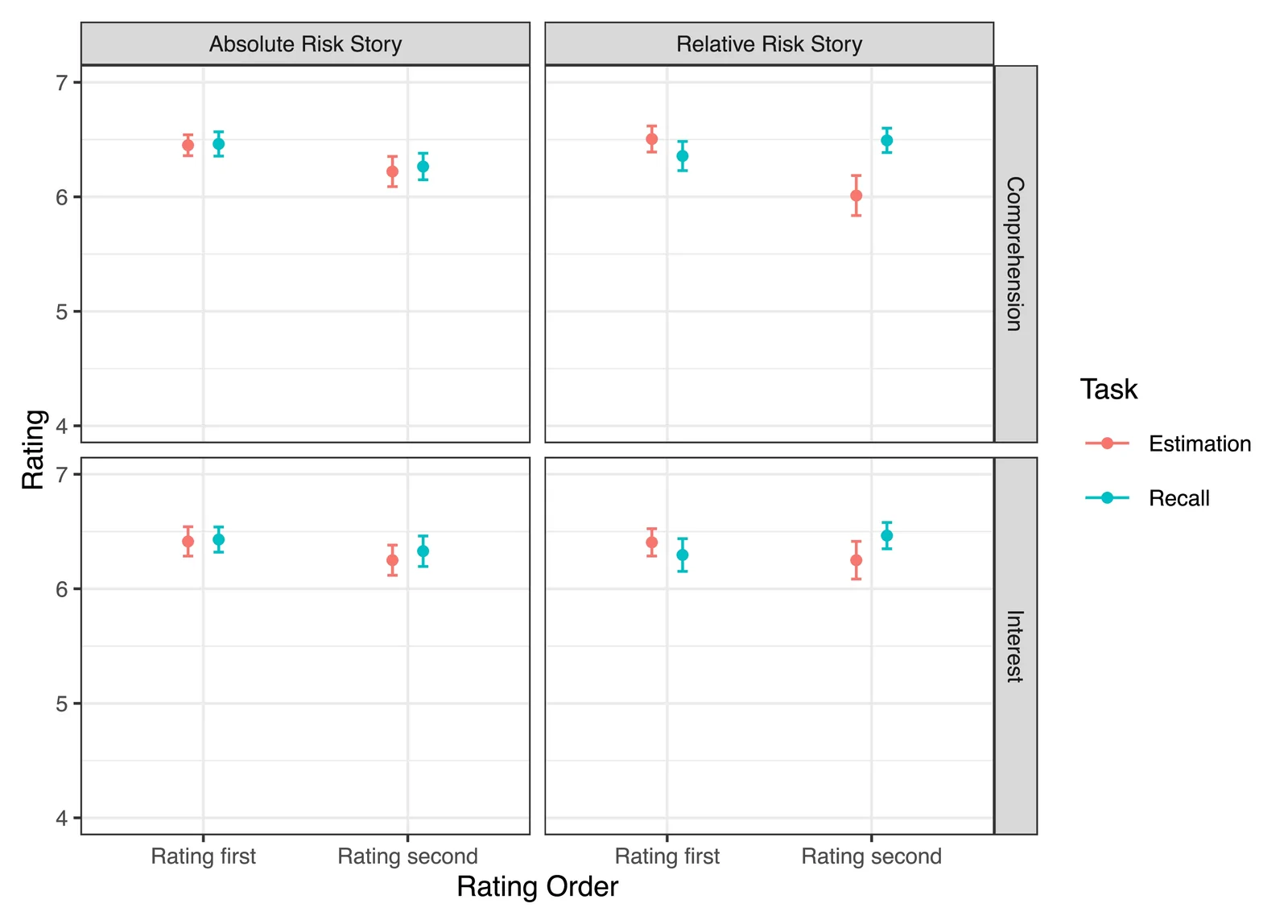
Top panels: comprehension ratings broken down by story type, rating order and task type. The panels show whether participants read the absolute risk story (left) or relative risk story (right). The order variable on the *x*-axis indicates whether participants completed the risk task first (estimation or recall) or the rating first. Error bars are 95% confidence intervals. Note the *y*-axis is truncated at 4. The rating scale spanned 1–7 with 4 equivalent to a neutral rating, and above 4 a positive rating. Bottom panels: as above for interest ratings.

### 3.7. Exploratory analyses

In a series of exploratory analyses, we further examined the variability in people's baseline risk estimates and the extent to which their accuracy was affected by their education level, comprehension and interest in the story.^2^ We focus on participants who read the relative story and inferred the absolute risks.

One question was whether participants’ produced absolute estimates that conformed to the 50% decrease indicated by the news story. To examine this, we took each participant's difference between low and high estimates as a proportion of their high estimate (after excluding 30 participants who reported an increase rather than a decrease). Figure 7 shows the distribution of this ‘relative decrease’ metric, with a clear peak at 0.5 (the correct value).

**Figure 7.**
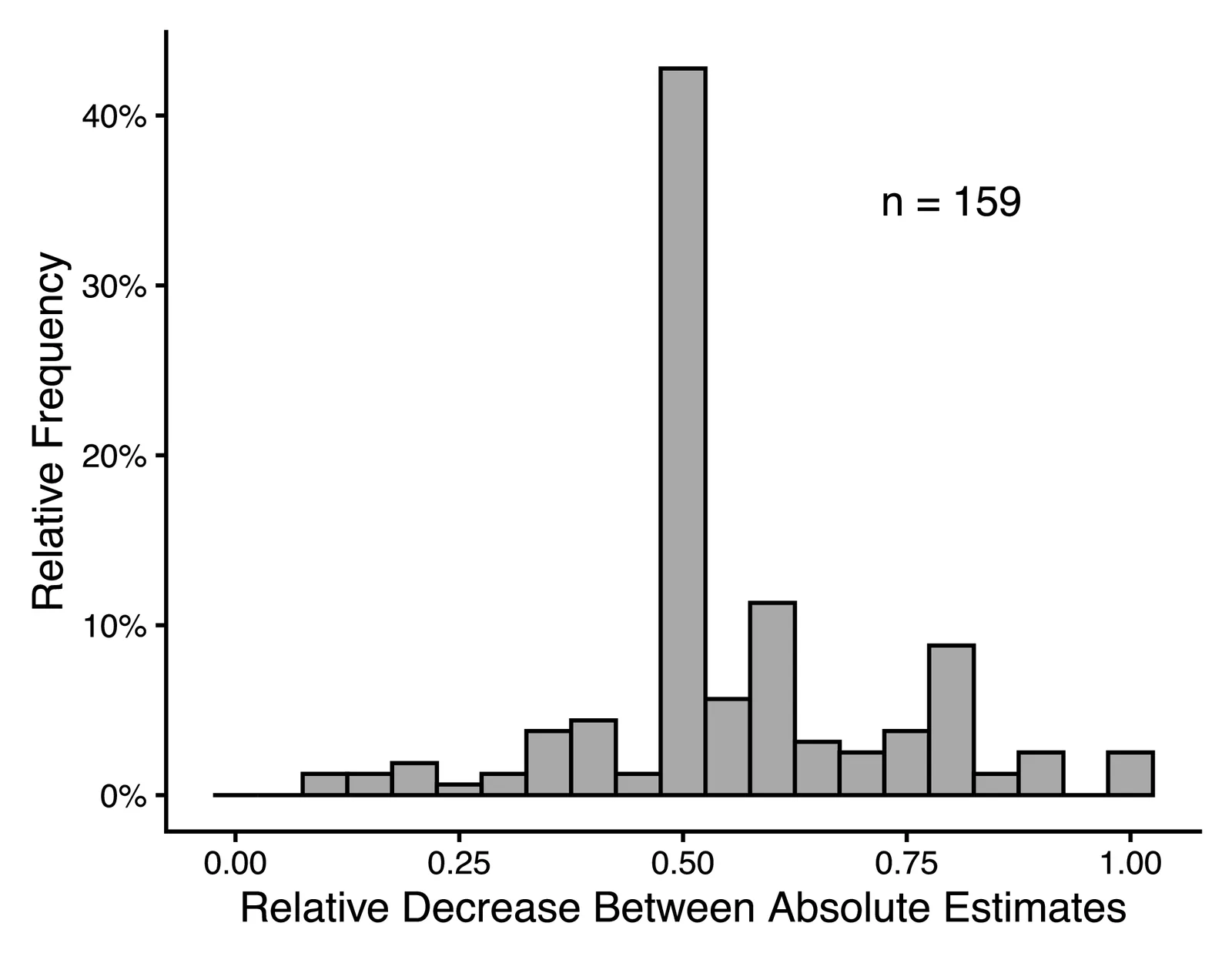
Histogram of the relative decrease from the high to low absolute estimates after reading the relative story. Calculated within-participant.

We then calculated the absolute difference between each participant's relative decrease and the normative value of 0.5, as a measure of the accuracy/coherence of their responses. This measure was not significantly correlated with participants’ estimates of the baseline risk, *ρ*(*N*  =  159) = 0.14, *p*  =  0.076, or with their self-reported comprehension, *ρ*(*N*  =  159) = −0.03, *p*  =  0.702.

Finally, we looked at whether people's accuracy in estimating the baseline risk (having read the relative story) was predicted by education, interest or comprehension. A linear regression was conducted to determine, if education predicted absolute error on baseline risk estimates. Education was coded as a numeric variable (0 = none, 1 = school, 2 = undergraduate, 3 = postgraduate). The results indicated that education was not a significant predictor of baseline risk estimates, *β*  =   − 1.24, *t*(187) = −0.87, *p*  =  0.385 (a regression with education coded as categorical predictor also showed no significant effects). Interest was not correlated with baseline risk absolute error *ρ*(*N*  =  189) = −0.08, *p*  =  0.285 and comprehension was also not correlated with baseline risk absolute error *ρ*(*N*  =  189) =   − 0.06, *p*  =  0.426.

## 4. Discussion

Participants in this study were visitors to the BBC news website who read a story about the association between mushroom consumption and reduced risk of mild cognitive impairment. The change in risk of MCI was either presented as a relative risk or in absolute terms. Participants who read the relative risk version and then estimated the absolute risks made far larger errors than those recalling the risks from the absolute risk version of the story.

Although readers of the relative risk version provided median estimates of the absolute risks that were close to correct, we think the most likely explanation of this finding is a wisdom of the crowd effect, whereby errors cancel each other out [17]. Despite the accurate group-level medians, the vast majority of participants (97%) were not correct in their estimates. It is also possible that this wisdom of the crowd result would not hold for much smaller or more obscure risks, for example a rare disease with a 1 in 10 000 base rate. Consequently, we would urge against assuming (even at the group level) that relative risks allow people to infer the correct absolute risks. At the same time, the correct group-level median suggests that readers were not wholly misled by the relative-risk framing. This is consistent with recent evidence suggesting that the public may be more resilient than sometimes assumed to at least some forms of media emphasis [18].

There was no difference in how participants rated the two formats in terms of how interesting they found the story. There was also no main effect on comprehension ratings, but there was an interaction such that comprehension ratings for the relative risk story were lower if participants were first asked what they thought the absolute risks were. If on the other hand, participants rated their comprehension before being asked about the absolute risks, there was no difference.

It seems likely that being asked about absolute risks after reading relative risks led participants to question their understanding of what they had read, hence the lower ratings. Given that the average reader of a news article is not typically asked follow-up comprehension questions, it seems reasonable to infer that neither story (in the absence of comprehension questions) would be rated as easier to understand than the other. These results are encouraging when it comes to advocating for the use of absolute risks in news stories; the absolute format was considered just as interesting and comprehensible as the relative format.

Participants who read the absolute format story were also asked to report the relative change. The modal response (10% reduction) was incorrect and suggests that people largely interpreted the question to be about the percentage point change (for which 10 would be the correct answer). The second most frequent response was a 50% reduction, which was approximately the correct relative change.^3^ This result highlights how easily confused these two metrics are. As noted in §1, previous research has shown that given a baseline risk and a relative change, people struggle to calculate the absolute change [9].

It is important to acknowledge some limits to the generalizability of the above findings. Perhaps most important is that we could not randomly sample from readers of the story, our participants were self-selecting. Therefore, the demographics of our participants may differ from those of the overall readership of the story. Our sample also skews old and educated relative to the general population. Participants who followed the link to our study probably read the entirety of the story and perhaps were more interested in it than the average person browsing through the news.

This study relates to one story and to a relative reduction in risk. We cannot know how well these findings generalize to domains other than health, different sizes of baseline risk, or to increases rather than reductions in risk. These are questions for further research and there are good theoretical reasons to think that these aspects might matter. The psychology of judgement and decision-making reveals that very small and very large risks are most subject to distortion in people's judgements, with small risks typically being overestimated and large risks typically being underestimated [20]. Furthermore, there is evidence that these effects are moderated by affect and numeracy [21].

A fall in risk may also be perceived differently from an equivalent rise in risk. The framing effect [22], for example, tells us that people are more likely to choose a treatment when it is framed in terms of survival rather than mortality [23] so a two thirds survival rate is not treated the same as a one third mortality rate. Indeed, there is evidence for this in doctors’ perceptions of absolute risks. One study of doctors found that an absolute mortality reduction was considered by 68% of participants to be effective, while the equivalent absolute increase in survival was only considered by 52% of participants to be effective [10]. One explanation for these effects is that the frame implies a recommendation, for example, highlighting mortality could be seen as advice to avoid a treatment [24].

The finding that people were on average correct necessarily depends on readers as a whole being able to make an unbiased estimate of the baseline risk. This could be affected by a number of factors. Our exploratory analyses suggest that none of education, interest or comprehension were associated with being accurate at estimating the baseline risk. However, the large majority of our sample rated their interest and comprehension very highly (see figure 5) potentially making it difficult to detect effects of these factors. Very common risks may be well-known in which case we would expect more precision and less benefit from the absolute format. Conversely, if there is a widespread misconception about how risky something is, we would not expect this to average out in a wisdom of the crowds type effect when readers are only given relative risks. A topic that readers find particularly distressing (perhaps childhood diseases) might also be expected to elicit biases in estimates given the aforementioned effects of emotion on risk assessment. Critically though, these are all limitations with respect to people being able to infer, on average, the correct absolute risk from only relative information. All of these issues can be mitigated simply by providing the absolute risks instead.

These limitations should be considered alongside several strengths. The study had excellent ecological validity, given that this was a real story live on the BBC news website and participants would have read it unaware of the study until they reached the end of the story. It is also worth highlighting that visitors to the site were randomized to see either the relative or absolute version. We can also be confident in the statistical results given the large sample size and pre-registered analyses.

Overall, the study is encouraging with respect to the benefits and feasibility of using absolute risks in news stories. The absolute format maintained comprehension and interest while reducing error in estimates of absolute risk. The literature on risk communication also provides strong support for the use of natural frequencies over percentages (i.e. 1 in 10 rather than 10%) [25,26]. Indeed, the use of absolute risks is now commonplace in guidance on how to communicate with patients in medical settings [27,28].

That said, the use of absolute numbers is not a blanket recommendation. There are cases where the absolute numbers may seem more sensationalist than the relative change. Unemployment figures, for instance, where the change in proportional terms may be more instructive than the absolute numbers (e.g. ‘unemployment rises by 0.1%’ versus ‘unemployment rises by 18 000’). There are also instances where a low absolute risk with severe consequences can be easily mitigated (e.g. wearing a seatbelt) and it might be appropriate to use the relative risk as a communication device [29].

Responsibility for how changes in risk are reported does not lie exclusively with media organisations. Researchers, university press officers, academic journals, journalists and editors all have incentives to maximize how interesting and impactful a finding is portrayed to be. One analysis of press releases by academic medical journals found only 55% included base rates when comparing two groups [30].

## 5. Conclusion

Absolute risks were remembered, understood and found equally as interesting as relative risks by an audience reading a real BBC news story. They also resulted in a much more precise perception of the actual absolute risk compared to those who read the story in a relative risk format.

## Data Availability

The data can be accessed here: https://doi.org/10.48420/32603361 [31].

## Footnotes

^1^
Note that the Wilcoxon test probes whether the data are symmetrically distributed about a specified value—in this case, the correct response. If the data are symmetrically distributed, a ‘significant’ result indicates that the observed median deviates from the specified value. However when, as in the current examples, the data are skewed, a significant result indicates the more general finding that the data are not symmetrically distributed about the specified value.

^2^
With thanks to an anonymous reviewer for suggesting these analyses.

^3^
It is worth noting here that 50% is sometimes over-represented when eliciting probability estimates from the public who can use it as a default value when uncertain [19].
